# Multi-Way Analysis Coupled with Near-Infrared Spectroscopy in Food Industry: Models and Applications

**DOI:** 10.3390/foods10040802

**Published:** 2021-04-08

**Authors:** Huiwen Yu, Lili Guo, Mourad Kharbach, Wenjie Han

**Affiliations:** 1 Chemometric and Analytical Technology, Department of Food Science, Faculty of Science, University of Copenhagen, Rolighedsvej 26, DK-1958 Frederiksberg C, Denmark; huiwen.yu@food.ku.dk; 2 Department of Plant and Environmental Science, Faculty of Science, University of Copenhagen, Højbakkegaard Alle 13, DK-2630 Taastrup, Denmark; 3 College of Water Resources and Architectural Engineering, Northwest A&F University, Weihui Road 23, Yangling 712100, China; 4 Research Unit of Mathematical Sciences, University of Oulu, FI-90014 Oulu, Finland; mourad.kharbach@hotmail.fr; 5 School of Science and Engineering, The Chinese University of Hong Kong, Shenzhen 518172, China; hanwenjiezhongshan@126.com

**Keywords:** near-infrared spectroscopy, food industry, chemometrics, multi-way analysis, applications

## Abstract

Near-infrared spectroscopy (NIRS) is a fast and powerful analytical tool in the food industry. As an advanced chemometrics tool, multi-way analysis shows great potential for solving a wide range of food problems and analyzing complex spectroscopic data. This paper describes the representative multi-way models which were used for analyzing NIRS data, as well as the advances, advantages and limitations of different multi-way models. The applications of multi-way analysis in NIRS for the food industry in terms of food process control, quality evaluation and fraud, identification and classification, prediction and quantification, and image analysis are also reviewed. It is evident from this report that multi-way analysis is presently an attractive tool for modeling complex NIRS data in the food industry while its full potential is far from reached. The combination of multi-way analysis with NIRS will be a promising practice for turning food data information into operational knowledge, conducting reliable food analyses and improving our understanding about food systems and food processes. To the best of our knowledge, this is the first paper that systematically reports the advances on models and applications of multi-way analysis in NIRS for the food industry.

## 1. Introduction

Near-infrared spectroscopy (NIRS) is a fast and powerful analytical tool. It has gained widespread acceptance in both the scientific community and industry. NIRS uses the near-infrared region of the spectrum, commonly defined from 780 nm to 2500 nm, where most absorption bands are molecular overtones or combination vibrations bands [1]. Compared with other spectroscopy methods, NIRS is deemed to be more attractive by virtue of its non-destructive nature, high speed of analysis, ease-of-use and low cost [2]. It is widely applied in various fields, such as environment [3,4], agriculture [5,6], food [7,8], pharmaceutical [9,10], clinical medicine [11,12], and remote sensing [13,14]. As one of the dominant vibrational spectroscopies in the food industry, NIRS is extensively used for food classification, component characterization, quality evaluation and process control, where its effectiveness for fingerprinting food materials and analyzing different critical parameters has been proven [15].

The rapid development of chemometrics during the past decades is progressing and advancing a lot of scientific areas which are highly overlapped with analytical science, including food science [16]. Combined with an instrumental analysis, the use of chemometrics tools leads to more advances in understanding food products and food systems [17]. However, some challenges arise in food science when entering a digital and instrument-heavy area. One of the biggest challenges is to effectively explore and model the complex and large data sets; NIRS data are one such data source. It is well known that NIRS suffers from the problems of nonlinear behavior, the scatter effect and broad overlapped bands, as well as the artifact effect, such as temperature disturbances, which easily lead to models with bad predictions and challenging interpretations. Previous studies have shown that rearranging data with nonlinear behavior into higher-order arrays can be a promising way to improve the predictions of the chemometrics model [18]. Dealing with this kind of high-order array requires high-order chemometrics tools which are complex but more promising for producing satisfying results. Meanwhile, the widely used two-way chemometrics tools for modeling NIRS related data, such as a principal component analysis (PCA) [19,20] and partial least square (PLS) [21,22], have different drawbacks in practice. For example, they have difficulty in predicting the new samples with a slightly different nature (e.g., due to unexpected temperature variations), and sometimes the interpretation of the models’ results can be very difficult because of the rotation freedom [20]. Moreover, the model performance can be easily affected if too much redundant information existed in the modeled spectra. Even though variable selection methods can be beneficial for improving models to some extent, the risk of overfitting cannot be ignored in this approach. Advanced chemometrics tools such as multi-way analysis methods could thus be considered by food technologists and researchers for the purpose of analyzing complex NIRS data and establishing valuable models with more flexibility, simple interpretations, and robust results [23].

In many scientific fields, a multi-way data analysis is popular and frequently appears under the name tensor analysis [24]. The multi-way analysis originated from the 1960s in social science [25,26]. Subsequently, many chemometricians contributed to the development of multi-way models and solved many issues in this area [27]. For example, Wold et al. [28] provided the initial idea of N-way partial least square regression (N-PLS) in the 1980s and illustrated it with chemical mixture data recorded from liquid chromatography and ultraviolet spectrometry. Bro [29] further developed the real N-PLS model for both three-way and higher order arrays, and illustrated the algorithms with an example from the sugar industry. The application of the multi-way analysis for solving food science problems is relatively new. It has, however, shown a great deal of success in analyzing complex food data and solving a wide range of food-related problems [30,31,32]. Owing to the so-called second order advantage [33], most of the multi-way analysis models have the ability to extract hidden information from complex data, finding the latent relationship between variables, while avoiding using a large number of different calibration sets [34]. For instance, the combination of multi-way analysis with spectroscopy can be used for the real-time reaction process monitoring of compounds of interest for a complex food system. It was reported that the combination of NIRS, multi-way chemometrics and process knowledge is taking place in process analytical technology (PAT) [23]; that is to say, multi-way analysis provides a useful tool for analyzing complex process data [35]. Moreover, other “higher-order advantages” of multi-way analyses, e.g., increasing sensitivity when the data dimension increases and the ability to solve high collinearity, are also beneficial for solving many practical problems [36].

In this paper, we focus on models and applications of multi-way analysis combined with NIRS for both food products and food process analyses. The contents of this paper are organized as follows. The second part explains the used notations and abbreviations. The third part describes the multi-way models which were used for analyzing NIRS data, as well as the advances, advantages and limitations of different models. The preprocessing techniques for multi-way analysis for analyzing NIRS data are briefly described in the fourth part. In the fifth part, the applications of multi-way analysis in NIRS for the food industry are reviewed in terms of food process control, quality evaluation and fraud, identification and classification, prediction and quantification, and image analysis. The available software and algorithms for multi-way analysis are introduced in the sixth part. The conclusions are formulated in the last part. To the best of our knowledge, this is the first paper that systematically reports the advances on models and applications of multi-way analysis in NIRS for the food industry.

## 2. Notation and Abbreviations

In order to avoid confusion, we will use the conventional and standard notations from the multi-way analysis community. Consistent with the notation used in Kiers [37], we use non-bold italic letters, boldface lower-case italic letters and boldface upper-case letters to denote the scalars, vectors and matrixes, respectively, e.g., A is a matrix, $a_{f}$ is the *i*th column of matrix A and F is the number of components of the model. A three-way array is denoted by $\underline{X}$. Each dimension of the three-way array is called a mode. The element of the three-way array is denoted by $x_{ijk}$ where *i*, *j* and *k* mean the indexes belonging to each of three modes of $\underline{X}$. The symbol ⊗ is used to represent the Kronecker product, while * denotes the Hadamard product [38]. All the used abbreviations in this paper are shown in Table 1.

## 3. Multi-Way Models

### 3.1. N-PLS

N-way partial least squares (N-PLS) is a regression algorithm combining tri-linear decomposition and the classical PLS. In fact, it is an extension of the two-way PLS model to the multi-way case. The real N-PLS model was proposed by Bro [29] in 1996, which can be expressed as:

(1) $$X=T{\left(W^{K}⊗W^{J}\right)}^{T}+E_{X}$$

where the I×JK matrix X is the unfold version of three-way array $\underline{X}$ (I×J×K), $W^{J}$ and $W^{K}$ are the weights matrixes of the second and third modes, T is the score matrix of the first mode, and $E_{X}$ is the residual matrix. Imagine Y contains the dependent variable, then $Y=UH+E_{Y}$, where U and H are the scores matrix and loading matrix of Y, and $E_{Y}$ is the residual for Y. The aim of the N-PLS model is to find the weight matrixes $W^{J}$ and $W^{K}$ that maximize the covariance between U and T. It is necessary to point out that this initial N-PLS model was limited by some problematic issues such as the perfect fit problem, uniqueness issues and impossible validation assessments of the parameters [39]. In order to fix these problems, Bro et al. [39] later proposed a modified N-PLS model by introducing a core array in the model of $\underline{X}$, and the new version of N-PLS model is widely used in different software today. The modified N-PLS model can be written as:

(2) $$X=TM_{X}{\left(W^{K}⊗W^{J}\right)}^{T}+E_{X}$$

**Figure 1:**
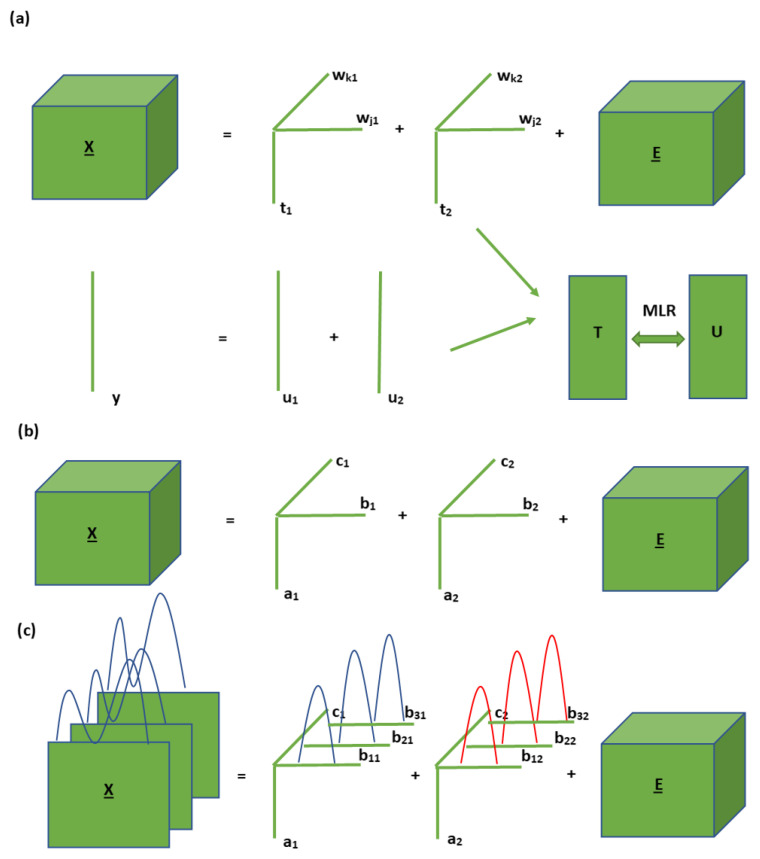
Graphical representation of the modeling process of: (**a**) N-way partial least square regression (N-PLS); (**b**) parallel factor analysis (PARAFAC) and (**c**) parallel factor analysis 2 (PARAFAC2) on three-way data with two components.

The difference with the old N-PLS model is the core array $M_{X}$. It is the matricized core array of size F×F×F, and it equals $T^{+}X{\left({\left(W^{K}\right)}^{+}⊗{\left(W^{J}\right)}^{+}\right)}^{T}$, where + means the Moore–Penrose pseudo inverse. The new N-PLS model produces the same predictions as the original N-PLS model since only the way for modeling $\underline{X}$ was optimized and nothing happened for the prediction part in the modified N-PLS model. More details about the calculation of the parameters can be found in Bro et al. [39]. The so-called N-way partial least square regression-discriminant analysis (N-PLS-DA) model is just the discriminant version of the N-PLS model. In N-PLS-DA, the dependent variable holds the class information, where each response variable is defined as a dummy variable with different values indicating a different category. A graphical representation of the reported multi-way models for three-way array with two components is shown in Figure 1.

Compared with the two-way PLS model, the N-PLS model retains the multi-way information of the data, avoids the huge number of parameters caused by unfolding the multi-way array and the difficult model interpretation caused by the confounding of modes. It is capable of identifying multi-way data patterns and complex feature correlation. Apart from these advantages, N-PLS also has advantages in better modeling accuracy, robustness to noise, stabilized solution, increased predictability, etc. [40]. Some progress concerning the algorithm has been accomplished in recent years. Faber and Bro [41] investigated two methods for estimating the standard error of prediction in N-PLS models by calculating the estimates of all error variance and calculating an estimate of the standard deviation of the measurement error in the reference. In order to conduct variable selection for multi-way array with two spectral dimensions, Favilla et al. [42] extended the Variable Importance in Projection (VIP) method to the N-PLS model, which was illustrated to work well. Recently, Biancolillo et al. [43] proposed a sequential and orthogonalized N-PLS (SO-N-PLS) algorithm for analyzing multi-way data blocks by combining N-PLS and the so-called Sequential and Orthogonalized-PLS, and better model performances on small data sets and noisy data were achieved by this algorithm. Even though N-PLS is an attractive multi-way regression algorithm, it is important to point out that N-PLS models do not have second order advantages since they work under the same premises as ordinary PLS. This means that N-PLS cannot handle new interferences that were not in the calibration set. Furthermore, proper variable selection and preprocessing procedures are strongly recommended when using N-PLS for analyzing NIRS data in practice. Modeling the most interesting region by multi-way methods can always increase the knowledge of the studied system.

### 3.2. PARAFAC (Parallel Factor Analysis)

PARAFAC was first proposed by Harshman [44] in 1970. In the same year, Carroll and Chang [45] also proposed an identical model called Canonical Decomposition (CANDECOMP) in the context of multidimensional scaling. Since the name CANDECOMP is more common in other fields than in chemometrics, we will use name PARAFAC in this paper. PARAFAC can be regarded as an extension of PCA but with many other advantages for decomposing multi-way arrays [46]. Instead of containing a score vector and a loading vector in a component like in PCA, PARAFAC decomposes the data into a set of tri-linear components and each component is composed of three vectors representing the three modes, respectively. For a three-way array, the model for PARAFAC can be expressed as:

(3) $$X_{k}=AD_{k}{\left(B\right)}^{T}+E_{k},k=1,\ldots ,K$$

where $X_{k}$ is a I×J matrix denoting the *k*th slab of three-way array $\underline{X}$, and the dimension of the three-way array $\underline{X}$ is I×J×K. Assuming F is the number of components of the PARAFAC model, A is a I×F matrix for the first mode, B is a J×F matrix for the second mode, $D_{k}$ is a F×F diagonal matrix in which the diagonal elements are the *k*th row of matrix C and represent the profiles of F components for the *k*th observation of the third mode, and $E_{k}$ is the residual matrix with the same dimension of $X_{k}$. From a perspective of vectors, the PARAFAC model can be also expressed using the Kronecker product:

(4) $$\underline{X}=\overset{F}{\underset{f=1}{\sum }}a_{f}⊗b_{f}⊗c_{f}+\underline{E},f=1,\ldots ,F$$

where $a_{f}$, $b_{f}$, $c_{f}$ denotes the *f*th column vector of loading matrixes A, B and C respectively, and $\underline{E}$ is the three-way residual array with the same dimension as $\underline{X}$.

One of the most attractive advantages of PARAFAC is the uniqueness of the solution [47], which means that the PARAFAC model cannot be rotated without a loss in fit. For PCA, it is well known that the inherent rotation freedom problem makes either external information or post-rotations necessary for the purpose of accurately identifying pure spectra. This is not the case in PARAFAC. If the data have a tri-linear structure and the noise in the data is appropriate, PARAFAC will always estimate the unique solutions (e.g., pure spectra) when using an appropriate number of components [46]. From a mathematical perspective, Kruskal [48] suggested the appropriate F should fulfill the condition of $k^{A}+k^{B}+k^{C}\geq 2F+2$ in order to get the unique solution, where $k^{A}$, $k^{B}$ and $k^{C}$ are the *k-ranks* of matrixes A, B and C, respectively. Regarding more practical details about how to select the appropriate number of components for the PARAFAC model, we recommend to look into the paper written by Bro and Kiers [49]. Like many other multi-way models, PARAFAC is also less sensitive to the noise in the data and produces models with less complexity than two-way chemometrics methods; a detailed illustration regarding this can be found in Bro [46]. Furthermore, PARAFAC is also capable of analyzing multi-way data with large amounts of miss values. Tomasi and Bro [50] proposed that incorporating the ALS (alternating least squares) procedure with a single imputation or implementing the Levenberg–Marquadt procedure in the PARAFAC algorithm can deal with the data with a large number of missing values. Besides the aforementioned benefits, PARAFAC is also advantageous in its simple model structure, yielding more adequate and interpretable models, and dealing with a big and complex dataset [23,51].

Even though PARAFAC has gained extensive acceptance nowadays, there are still some issues hampering the use of PARAFAC in practice. For example, multi-way analysis and data arrangement experiences are needed; such knowledge can be challenging for people who are accustomed to using only two-way chemometrics tools. Apart from this, algorithm problems are maybe another major concern. For instance, slow convergence occurs especially when facing seriously collinear data. The two-factor degeneracy problem, which means two components become almost identical but with opposite signs, is also a practical concern. In order to improve the convergence speed of the conventional PARAFAC-ALS algorithm, a wide range of remedies have been developed, such as line search [52], enhanced line search [53], extrapolation with optimized step size and search direction [54] and compression [46]. Besides the ALS algorithm, some alternatives for calculating PARAFAC models have also been proposed, including the all-at-once algorithm [55], the hierarchical conjugate gradient algorithm [56], a random gradient algorithm [57,58], the fast damped Gauss–Newton algorithm [52], etc. These new alternatives were reported to have better convergence speed for many real cases. When fast convergence is required, these algorithms are valuable for exploration and test in the specific case. Regarding the two-factor degeneracy problem, it happens mostly from using too many components or because the established PARAFAC model is not appropriate for the data. Generally, the Tuckers congruence [59] and components plot can be used to detect the possible degeneracy problem. It was also reported that imposing some constraints on the PARAFAC model was beneficial for avoiding the degeneracy problem to some extent [60].

### 3.3. PARAFAC2 (Parallel Factor Analysis 2)

PARAFAC2 was also proposed by Harshman [61] in the 1970s. It is an important extension of the PARAFAC model because it allows the loading matrix in one mode to be shifted or have different lengths for different entities in one mode. Following Kiers et al. [62], the PARAFAC2 model can be written as:

(5) $$X_{k}=AD_{k}{\left(B_{k}\right)}^{T}+E_{k},k=1,\ldots ,K$$

where $X_{k},\;A\;\mathrm{and}\;D_{k}$ are defined in the same way as in the PARAFAC model. The difference is that the second mode matrix B of PARAFAC is changed into $B_{k}$ in PARAFAC2, which means there is a specific and individual $B_{k}$ for each *k* of the—in this case—third mode. Thus, the strict tri-linearity assumption is actually relaxed in one mode in the PARAFAC2 model. Compared with the assumption of PARAFAC which is requiring B to keep the same shape for each *k*, only the cross products of $B_{k}$ are constrained to be constant for each *k* in the third mode in PARAFAC2 [62]. This less strict constraint was illustrated to be a precondition for obtaining the uniqueness in PARAFAC2 by Ten Berge and Kiers [63]. The constant cross product property is obtained by making $B_{k}=P_{k}G$, where $P_{k}$ is an orthogonal matrix with dimension of J×F thus $P_{k}^{T}P_{k}=I$ and G is a common matrix with dimension F×F. Details about the mathematical proof can be found in the initial paper [62]. Hence, $P_{k}$ is concerned with the uniqueness for each observation in the varying mode (shift or different length) while G is concerned with the observations [64]. Understanding the uniqueness and the model assumption of the PARAFAC2 model is of vital importance since it will directly affect whether it is applied in a proper way for real data.

Owing to the advantage of the less strict tri-linearity requirement, which is often more realistic in the real world, PARAFAC2 is widely applied for solving a wide range of complex data analysis problems, e.g., the problem of varying batch trajectories in food process analyses (different batches have different numbers of NIRS spectra) or analyzing complex NIRS image data. As an important multi-way analysis model, PARAFAC2 has all the advantages of the general PARAFAC model. One of the challenges of the PARAFAC2 algorithm is how to impose the non-negativity constraints on all three modes. For the conventional PARAFAC2-ALS algorithm, it is not possible to impose a non-negativity constraint on the shifted mode, thus only two modes can be constrained. Recently, Cohen and Bro [65] proposed a flexible PARAFAC2 algorithm and made it possible to impose non-negativity constraints on all the three modes by casting the PARAFAC2 model as a coupled matrix factorization model. For the applications where all the modes are required to be non-negative, this flexible PARAFAC2 algorithm will be of great value. Furthermore, a core based PARAFAC algorithm has also been proposed recently with a possibility of imposing non-negativity constraints [66]. However, strict non-negativity cannot be guaranteed in this algorithm since the transformation matrixes operate on orthogonal factor matrixes. Another practical concern is the local minima problem. The PARAFAC2 decomposition is inherently a non-convex optimization problem, and thus it is a NP-hard problem [67] with one of its aspects being the local minima. A useful remedy for avoiding the local minimum is to repeat the PARAFAC2 model calculation for several times, then choose the model with the best fit as the global minimum model. However, it is very ad hoc. More explorations from the algorithm perspectives will be important for avoiding such problems. The convergence speed is also an issue for the PARAFAC2 algorithm. A recent research of Tian et al. [68] reported the effects of different line search strategies for accelerating PARAFAC2-ALS convergence, and proposed an acceleration procedure called geometric search which was faster than the non-accelerated PARAFAC2-ALS algorithm. However, seeking the efficient alternatives of the ALS algorithm for estimating PARAFAC2 model is still needed for the future.

### 3.4. Other Multi-Way Models

There are many alternative multi-way analysis models available for analyzing multi-way dataset. For instance, Tucker3 is a multi-way model of the Tucker family, also called the three-mode principal components analysis [25,69,70]. Compared with the PARAFAC model, a super-diagonal core array exists in Tucker3 models. It basically compresses all three modes of the data so that the main information can be summarized by a few components for each mode. Therefore, the number of components in a Tucker3 model can be different for each mode. It is important to notice that the unique and best subspace can be found by the Tucker3 model, while the decomposed loadings are generally not unique due to the rotation freedom problem. Another promising multi-way model is alternating tri-linear decomposition (ATLD) [71]. It was used for decomposing three-way data but was shown to have faster convergence than the PARAFAC-ALS algorithm in the original paper [71]. It was also based on the ALS principle but with an improved iterative procedure using the Moore–Penrose generalized inverse with a singular value decomposition. Limited by the length of this paper, only simple descriptions about these models are introduced here. More details about many other different multi-way models can be found in the reference [23,72].

## 4. Preprocessing Techniques

Preprocessing plays an important role in chemometrics; however, it is more complicated when used for a multi-way array compared with two-way cases [60]. Some studies have been done in terms of centering and scaling for a multi-way array [46,72,73,74]. Basically, three ways of centering can be performed on a multi-way array, including single-centering, double-centering and triple-centering [46]. Single-centering can be done by unfolding the three-way array into a matrix, and then centering the matrix as in an ordinary two-way chemometrics method, such as PCA: for example, unfolding the I×J×K array into I×JK matrix and centering it across the first mode. People can also center the data across one of other modes depending on the specific problem. Double-centering means two modes of the three modes are centered by first centering one mode; then, the outcomes of this centering are centered. In triple-centering, centering across all the three modes at a time will be performed. As stated by Bro et al. [60], single-centering is the only appropriate choice for performing centering on a multi-way array in order to fulfill the assumptions of multilinear models, e.g., the PARAFAC model. Regarding scaling, the assumptions of multilinear models also need to be considered. Since scaling column-wise (as in centering) will distort the underlying trilinear structure of the data [74], it is necessary to scale the whole slab within a specific mode instead of the columns. Several issues need to be taken into account when performing scaling on multi-way array. One of them is that scaling one mode will always affect the scale of other modes, which means that scaling within several modes can be more complicated. The amount of noise and unsystematic variation in the data may be increased after performing scaling on some types of multi-way data [74]; hence, centering across one mode or scaling within one mode is always the straightforward way to preprocess the multi-way array. More details about centering and scaling for a multi-way array can be found in the aforementioned references. For multi-way NIRS data, the conventional preprocessing techniques for NIR spectra can be used before rearranging the two-way NIR spectra into the multi-way array. Scatter correction and derivatives methods are the most widely used preprocessing techniques for NIR spectra. MSC [75], Inverse MSC [76], Extended MSC [77] and SNV [78] are commonly used for scatter correction, while first or second order Savitzky–Golay derivatives and Norris–Williams derivatives [79] are the widely used spectral derivative techniques. Moreover, the interval and combined versions of different preprocessing techniques are also frequently used in practice. For more details regarding parameters settings, the effects comparison and the methods selection for different preprocessing techniques for NIR spectra, we recommend the specific preprocessing references [80,81].

## 5. Applications of Multi-Way Analysis and NIRS in Food Industry

### 5.1. Process Analysis and Control

Food industrial processes and food productions often involve multi-way data. A number of reports have shown the great potential of multi-way analysis tools for analyzing high dimensional and complex food process data [82,83,84,85]. NIRS is widely applied in the process analysis, and a multi-way data analysis coupled with NIRS is gaining more and more acceptance in the food industry. Allosio-Ouarnier et al. [86] used PARAFAC to investigate the variation of barley during the malting process by analyzing the three-way array of wavelengths × batches × malting time. The batch difference and sample differences within a batch were both successfully observed. In order to confront the challenge of industrial complexity, Liu et al. [87] developed an industrial process analysis method by combining on-line NIRS and ATLD. The industrial process could be monitored by observing the variation of common property extracted from the ATLD model in different batches, and an application of tobacco production indicated that the multi-way analysis, such as ATLD, was more capable of extracting the intrinsic information hidden in the NIR spectra. This achieved a better performance in the process analysis compared with the conventional two-way analysis tools. Furthermore, Nielsen et al. [88] also applied an analysis of common dimensions and specific weights (COMDIM) [89] to analyze the NIRS data of wheat flours and argued that their proposal was a powerful tool for process control in the flour mill. The authors of this paper said that multi-way analysis was advantageous in simultaneously considering the variation in both the particle size and chemical data, while the two-way PCA analysis was insufficient. By doing so, different quality parameters could be optimized during the flour production. Temperature is a critical factor that cannot be ignored in NIRS in the process analysis. Peinado et al. [90] used PARAFAC-MLR to model the batch process of the in-line NIRS dataset of water and ethanol by considering temperature information as an additional parameter in the tensor. Even though the dataset has simultaneous changes in temperature and chemical composition, the physical–chemical changes happening in the evolving systems were extracted successfully by the PARAFAC model. The proposed tensor-based strategy was more attractive than PCA or PLS, and it was recommended to be implemented in various industrial applications. In the scientific literature, different multi-way analysis models have also been compared for food process analyses. Lillhonga and Geladi [91] monitored the fermentation of food waste mixture batches over time by combining multi-way analysis models and NIRS. They showed that PARAFAC and Tucker3 produce common time profiles for all the batches and that the half-lives can be estimated successfully, while PARAFAC2 yields an individual time profile for each batch with a little more noise and convergence problems. Vigni and Cocchi [92] studied the formulation effects on wheat flour during a leavening process by using PARAFAC and N-PLS. The mixtures’ differences regarding the leavening time were detected by the PARAFAC decomposition of the three-way NIRS dataset. The established N-PLS model on baked bread parameters successfully found the relationship among flour formulation, leavening time and the final product. The multi-way analysis was proven to be an effective tool for monitoring the industrial leavening process of wheat flours.

### 5.2. Fraud and Quality Evaluation

NIRS is widely used for the purpose of food quality evaluation and fraud detection [93,94,95]. Many studies have reported the effectiveness of the combination of NIRS and the multi-way analysis in dealing with such issues in the food industry. For example, the accurate information of adulterants in milk was generally difficult to be captured by two-way chemometric tools because of the weak and overlapped absorption bands of the adulterants [96]. Yang et al. [96] successfully used N-PLS to model the 2D NIR spectra data of melamine adulterated milk. By comparing with the PLS model results on 1D NIR spectra, the multi-way model was deemed to be more accurate and robust since its average relative error is only 22.9% while the average relative error of PLS model is 122.4%. Recently, some studies have reported the effectiveness of the multi-way analysis and NIRS in the oxidative stability evaluation of oil. Rosa et al. [97] reported that PARAFAC coupled with NIRS was powerful for evaluating the protective effect of plant-based substances and synthetic antioxidants against oxidation in soybean oil. The NIRS data were rearranged into a three-way array of samples by temperature by wavenumbers. They concluded that the proposed method was a simple and fast way to achieve the anti-oxidation evaluation for soybean oil. Furthermore, PARAFAC coupled with NIRS was also used for evaluating the degradation of thermal rice oil by Rosa et al. [98]. The oxidative stability of rice oils was evaluated in a fast way by combining the multi-way analysis and NIRS. Moreover, Favilla et al. [42] used NIRS and the N-PLS model to accurately model the critical properties of bread loaves. The flour performance of the leavening phase was then successfully and accurately predicted.

### 5.3. Identification and Classification

Identification and classification are important application fields of NIRS and chemometrics [99], and are also used for the consideration of the multi-way analysis. Cui et al. [100] compared the effects of M-PCA, ATLD and PARAFAC on both three-way and four-way temperature-dependent NIRS data. Their results showed that ATLD and PARAFAC were capable of capturing the spectral variation information for each component, while the loadings of M-PCA contained the mixed spectral information of all the components, even though it explained the variance of whole data. The authors concluded that the multi-way analysis model can determine the chemicals of aqueous solutions and it can be the best way for analyzing temperature-dependent NIR spectra. In order to find differences between the cultivars and localities, Geladi and Manley [101] used PARAFAC to analyze the three-way NIRS data of wheat flours and made a comparison with the two-way chemometric models. They showed that PARAFAC can detect the variation information of protein content and hardness values, which were associated with localities and cultivars separately, and merging the information of the cultivars and the localities in one-way and then building PCA or PLS models are not proper for their case. Allosio et al. [102] reported PARAFAC was useful for separating barley batches according to the malting process. Such classifications can be achieved by connecting the decomposed time mode profiles with wavelength mode information expressed as NIR spectral intensities. The authors state that PARAFAC had great potential in analyzing the time series NIR dataset. Recently, the multi-way analysis has been applied to the classification of milk. Yang et al. [103] combined N-PLS-DA and 2D NIR correlation spectra to illustrate the feasibility of classifying normal milk and tainted milk. They compared the effects of the N-PLS-DA model on 2D IR/IR, 2D NIR/NIR and 2D IR/NIR spectra using the correct classification rate (CCR). The results showed that N-PLS-DA models on 2D IR/NIR spectra can provide the best classification of milk with a CCR of 96.1%.

### 5.4. Prediction and Quantification

Multi-way analysis was also used for quantification and prediction purposes in the food industry [104]. In particular, the multi-way analysis coupled with NIRS has been successfully applied in many practical cases by virtue of better model predictive performance and the ability to simultaneously analyze chemical and physical variations [105,106]. Letíci et al. [107] used N-PLS models to predict the content of limonene and water in the spray-dried systems by analyzing the three-way NIRS data. The temperature variation can be modeled successfully and the prediction error of N-PLS model is only 0.2%, which was small compared with that of the two-way model. The conventional two-way NIRS calibration model faces many challenges because of its weak robustness when it is applied for analyzing the samples with a large amount of water [108]. To circumvent such a challenge, Peng et al. [108] developed a hybrid algorithm called wavelet packet transform orthogonal signal correction N-PLS (WPNOSC-N-PLS) by applying a wavelet packet transform and orthogonal signal correction into the N-PLS model. In combination with NIR, WPNOSC-N-PLS accurately determined the main components in the concentration of milk even in the presence of temperature variation interference. The authors argued that WPNOSC-N-PLS can provide better models with better precision and robustness, and it is attractive in solving a wide range of multidimensional problems. Recently, the multi-way analysis and NIRS has been applied to the component quantification in corn. Zhang et al. [109] reported the application of N-PLS on self-constructed three-dimensional NIR spectra which can capture accurate quantitative information of four components (moisture, oil, protein, and starch). Compared with the two-way PLS model, the proposed multi-way models achieved better predictive performance for the target compounds with a lower RMSEP (root mean square error of prediction). Benefiting from the multi-way advantage, N-PLS coupled with 3D NIR spectra was deemed to be a rapid and robust tool for the accurate quantification of food compounds.

### 5.5. Hyperspectral Image Analysis

Very little work has been done on a dedicated multi-way analysis for analyzing NIR image data even though multi-way methods have shown great potential for dealing with such types of structures. Folch-Fortuny et al. [110] used an N-PLS-DA model to detect the symptoms of *Penicillium digitatum* in citrus fruits by analyzing the multi-way features array of the NIR hyperspectral image. It was reported that almost 91% of citrus fruit infected by *Penicillium digitatum* can be successfully detected by the multi-way model at early stages of the harvest, which will be of great importance for automating fruit sorting systems so that infected citrus fruits can be expelled before affecting the normal fruits. Yang et al. [111] developed a combination system using a NIR hyperspectral image, wavelet transformation and N-PLS to predict the total viable count (TVC) of spiced beef during storage. The three-way array was organized as spiced beef samples by wavelength variables by the wavelet detail coefficient, then it was concluded that the three-way tri-linear data structures yield a more accurate model with better interpretations of TVC than the two-way data. The N-PLS model produced a better prediction and lower errors. Recently, PARAFAC2 was also applied for analyzing complex NIR image data and showed a satisfactory performance. For instance, Alexandrino et al. [112] monitored multiple solid-state transitions of lactose by analyzing the temperature series NIR hyperspectral image using PARAFAC and PARAFAC2 models. Their results showed that the PARAFAC model cannot work well in that case since the pixels data did not conform to the tri-linearity assumption strictly, while PARAFAC2 extracted the profile of the compounds successfully. This allowed one mode of the three-way data to be shifted. PARAFAC2 was recommended to be the best for analyzing the series hyperspectral image data. Obviously, the NIR hyperspectral image analysis is a promising technology for solving practical problems in the food industry. However, some inherent drawbacks, such as high dimensionality [113], huge amount of acquired data [114] and considerably time consumption of the analysis [115], cannot be ignored. How to build efficient models for analyzing such a large number of NIR image data is a challenge [116]. By virtue of the multi-way advantages in handling large datasets, the application of multi-way tools will be beneficial for dealing with such tasks. As stated by Koljonen et al. [117], the multi-way analysis is a potential field of future research for the hyperspectral NIR image analysis. The representative applications of the multi-way analysis coupled with NIRS for analyzing food related data in terms of process analysis and control, quality evaluation and fraud, identification and classification, quantification and prediction, and image analysis are summarized in Table 2.

## 6. Software and Algorithms

During the past decades, a number of software packages have been developed for performing multi-way analysis. One of the original software is the N-way toolbox [119] for Matlab. Most of the multi-way analysis algorithms, such as PARAFAC, PARAFAC2, N-PLS, Tucker3 and DTLD, are available in this source. Users can easily impose a variety of constraints on these algorithms and run them with different initialization methods, such as SVD, random initialization and ATLD. Another software is CuBatch [35] which was originally built for the process analysis. It also contains many multi-way analysis algorithms including PARAFAC, Tucker models, etc. There are also some other open-source software implementations used for multi-way analyses from other communities. For instance, the Tensor toolbox developed by Kolda and Bader [120] is powerful for analyzing a wide types of tensor, including dense, sparse, and symmetric tensors. Phan et al. [121] also developed a tensor decomposition Matlab package called Tensorbox which contains various optimized algorithms for decomposing a tensor, such as the fast damped Gauss–Newton CP algorithm [122]. In addition to these free software, there is also a commercial software called PLS_Toolbox [123], widely used in the chemometrics community by virtue of its advantages in its easy-to-use, user-friendly interfaces and powerful visualization. All the aforementioned packages run under the Matlab environment. Recently, some multi-way analysis software packages running under the R environment have also been developed, such as ThreeWay [124] and multiway [125] packages developed by social science statisticians. Meanwhile, there are also some Python packages for multi-way analyses available, such as Tensorly [126] and TensorD [127]. The details of all the mentioned software are shown in Table 3, and simple introductions of different multi-way analysis algorithms can be found on the corresponding download websites.

## 7. Conclusions

The application of multi-way analysis combined with NIR spectroscopy is still located at the initial stage for the food industry. So far, what we have seen about the synergy between rapid spectroscopic sensors and data analytic technologies, which has revolutionized the food industry, is only the beginning. Even though NIR spectroscopy can rapidly obtain thousands of data points in a short time, the potential of these big data sets has not been fully investigated. Deeper statistical and chemometric knowledge is desired by the food technologists, owing to the challenges of the high complexity of food processes and food products. As an advanced chemometric tool, multi-way analysis not only shows powerful advantages in food process analysis, quality evaluation, determination of chemical composition and structure, food image analysis, etc., but also makes the analysis process greener with the help of green and smart “mathematical separation” [36], fulfilling the requirement of a sustainable food industry. Therefore, the combination of multi-way analysis with NIR spectroscopy will be a promising practice for turning food data information into operational knowledge, conducting reliable food analyses, and improving our understanding about food systems and food processes. For the future of the research, making the multi-way algorithms more efficient and less prone to numerical problems, such as local minima and two factor degeneracy, are the main numerical challenges. How to analyze larger amounts of data coming from the food industry process and food products is also a practical issue. Parallel processor computing will be valuable for alleviating such issues. Moreover, extending preprocessing techniques used for two-way chemometric methods for multi-way cases and dealing with systematically missing data are also important issues.

## Figures and Tables

**Table 1:** The meaning of used abbreviations in the paper.

Abbreviations	Explanations	Abbreviations	Explanations
NIRS	Near-infrared spectroscopy	N-PLS-DA	N-way partial least square regression-discriminant analysis
N-PLS	N-way partial least square regression	ATLD	Alternating tri-linear decomposition
PARAFAC	Parallel factor analysis	M-PCA	Multiway principal component analysis
PARAFAC2	Parallel factor analysis 2	PAT	Process analytical technology
Tucker3	A tensor decomposition method proposed by Tucker	MLR	Multiple linear regression
CP	The combination name of canonical decomposition and PARAFAC	COMDIM	Analysis of common dimensions and specific weights
SNV	Standard normal variate	MSC	Multiplicative scatter correction
PCA	Principal component analysis	PLS	Partial least square
DTLD	Direct trilinear decomposition	VIS/NIR	Visible-near-infrared spectroscopy
FT-NIR	Fourier transform near-infrared spectroscopy	FT-IR	Fourier-transform infrared spectroscopy

**Table 2:** Representative applications of multi-way analysis models coupled with near-infrared spectroscopy (NIRS) for analyzing food data.

Applications	Analyte	Data Arrangement	Preprocessing	Variable Selection	Multi-Way Analysis Algorithm	Spectral Range (Wavenumber or Wavelength)	Analytical Technique	Reference
Process analysis and control	Malt	Wavelengths × batches × time	Centering	Manual	PARAFAC	400–2500 nm	NIR	[86]
	Tobacco	Samples × variables × batches	Continuous wavelet transform	Not specified	ATLD	4000–12,000 cm^−1^	NIR	[87]
	Wheat flour	Samples × wavelengths/laser particle size/chemical × number of data matrices	SNV	Not specified	COMDIM	1100–2500 nm	NIR	[88]
	Food waste	Batches × time × wavelengths	Derivatives	Manual	PARAFAC, Tucker3 and PARAFAC2	905–1682 nm	NIR	[91]
	Water and ethanol mixture	Batches × wavelengths × temperature	Centering	Manual	PARAFAC	580–1090 nm	NIR	[90]
	Wheat flour	Mixtures × wavelengths × leavening times	Savitsky–Golay and SNV	Not specified	PARAFAC and N-PLS	1380–2250 nm	NIR	[92]
Fraud and quality evaluation	Milk	Samples × wavelengths × wavelengths	Fourier transformation	Manual	N-PLS	4000–10,000 cm^−1^	FT-IR	[96]
	Soybean oil	Samples × temperature × wavenumbers	Savitzky–Golay derivatives	Not specified	PARAFAC	900–1680 nm	NIR	[97]
	Rice oil	Samples × temperature × spectra	Baseline correction and Savitzky–Golay derivatives	Not specified	PARAFAC	900–1680 nm	NIR	[98]
	Bread	Leavening times × flour formulations × wavelengths	Savitsky–Golay derivatives and SNV	Variable importance in Projection	N-PLS	1380–2250 nm	NIR	[42]
Identification and classification	Ethanol	Temperature × wavelengths × samples	Continuous wavelet transform with a vanishing moment 2	Not specified	PARAFAC and ATLD	5500–12,000 cm^−1^	NIR	[100]
	Wheat flour	Locality × cultivar × wavelengths	Savitzy–Golay derivatives	Manual	PARAFAC	4000–10,000 cm^−1^	FT-NIR	[101]
	Milk	Samples × wavelengths × wavelengths	Mean-center	Manual	NPLS-DA	4000–10,000 cm^−1^	FT-IR	[103]
	Barley	Wavelengths × batches × time	Centering	Not specified	PARAFAC	1100–2500 nm	NIR	[102]
Prediction and quantification	Microcrystalline cellulose mixture	Concentration levels × wavelengths × compaction pressure levels	Centering, SNV, and Savitzky–Golay derivatives	Not specified	PARAFAC	1097–2200 nm	NIR	[105]
	Limonene and water	Samples × temperatures × wavelengths	Extended inverted signal correction, and direct orthogonalization	Interval-PLS	N-PLS	1100–2498 nm	NIR	[107]
	Milk	Samples × temperatures × wavelengths	Discrete wavelet packet transform and 3D orthogonal signal correction	Not specified	WPNOSC N-PLS	1100–2300 nm	FT-IR	[108]
	Corn	Spectral number × wavelengths × samples	Not specified	Not specified	N-PLS	1100–2498 nm	NIR	[109]
NIR image analysis	Citrus fruits	Fruit variety × features ×wavelengths	MSC and SNV	Permutation testing	NPLS-DA	650–1080 nm	VIS/NIR hyperspectral imaging	[110]
	Spiced beef	Spiced beef sample × wavelength variables × wavelet detail coefficient	Wavelet transform	Manual	N-PLS	400–1000 nm	VIS/NIR hyperspectral imaging	[111]
	Lactose	Pixels × spectra × time/temperature	Logarithmization, SNV and Savitzky–Golay derivatives	Hypertools [118]	PARAFAC and PARAFAC2	1000–1700 nm	NIR hyperspectral imaging	[112]

**Table 3:** Free and commercial multi-way analysis software.

Software	Running Environment	Multi-Way Analysis Algorithms	Website for Installation
N-way toolbox	Matlab	PARAFAC, PARAFAC2, N-PLS, Tucker models, GRAM, DTLD, etc.	http://www.models.life.ku.dk/nwaytoolbox (7 April 2021)
CuBatch	Matlab	PARAFAC, PARAFAC2, N-PLS, Tucker models, etc.	http://www.models.life.ku.dk/cubatch (accessed on 7 April 2021)
Tensor toolbox	Matlab	PARAFAC, Tucker models, Poisson tensor factorization, Generalized CP tensor factorization, Symmetric CP tensor factorization, etc.	https://www.tensortoolbox.org/ (accessed on 7 April 2021)
Tensorbox	Matlab	PARAFAC, Tucker models, Generalized Kronecker tensor decomposition, Tensor deconvolution, Tensor train decomposition, etc.	https://github.com/phananhhuy/TensorBox (accessed on 7 April 2021)
PLS_Toolbox	Matlab	MPCA, PARAFAC, PARAFAC2, N-PLS, etc.	https://eigenvector.com/ (accessed on 7 April 2021)
ThreeWay	R	PARAFAC, Tucker models, etc.	https://cran.r-project.org/web/packages/ThreeWay/index.html (accessed on 7 April 2021)
multiway	R	PARAFAC, PARAFAC2, Tucker models, etc.	https://cran.r-project.org/web/packages/multiway/index.html (accessed on 7 April 2021)
Tensorly	Python	PARAFAC, Tucker models, Tensor train decomposition, etc.	http://tensorly.org/dev/index.html (accessed on 7 April 2021)
TensorD	Python	PARAFAC, Tucker models, Pairwise interaction tensor decomposition, etc.	https://github.com/Large-Scale-Tensor-Decomposition/tensorD (accessed on 7 April 2021)
